# Critical-edge based tabu search algorithm for solving large-scale multi-vehicle Chinese postman problem

**DOI:** 10.1038/s41598-024-62992-2

**Published:** 2024-05-30

**Authors:** Jizhou Tang, Lili He, Yinghui Cao, Hongtao Bai

**Affiliations:** 1 School of Computer Science and Technology, Jilin University, Qianjin Street, Changchun, 130022 China; 2 Symbol Computation and Knowledge Engineer of Ministry of Education, Jilin University, Qianjin Street, Changchun, 130012 China

**Keywords:** Computational science, Computer science

## Abstract

The min–max multi-vehicle Chinese postman problem is an NP-hard problem, which is widely used in path planning problems based on road network graphs, such as urban road structure probing planning, urban road underground cavity detection planning, high-voltage line inspection planning, and so on. With the rapid increase in the number of nodes and connections of road network graph, the solution time and path equilibrium constraints pose new challenges to the problem solving. In this paper, we propose a critical-edge tabu search algorithm, CTA-kroutes, for solving the min–max multi-vehicle postman problem for large-scale road networks. First, the initial solution with balanced path lengths is obtained by segmenting the Eulerian paths; second, the critical edges are moved in the initial solution to construct the neighborhood solution, and the tabu search algorithm is used to find the optimal solution iteratively; and lastly, the solution optimization algorithm is used at the end of each iteration to de-duplicate and optimally reconstruct the current search result. Experiments show that the CTA-kroutes algorithm can effectively improve the equalization of multi-vehicle paths and its applicability to large-scale road networks.

## Introduction

The multi-vehicle Chinese postman problem (KCPP) is an important Arc Routing Problem (ARP) problem, which is a multi-vehicle version of the Chinese postman problem (CPP) ^1,2^. The KCPP problem involves finding k optimal closed paths within a network under specific constraints, with the requirement that each edge in the network is traversed by at least one path. Currently, scholars have studied for many different constraints, and the relevant constraints are: mixed road network constraints ^3^, capacity constraints ^4^ and time constraints ^5^. In the tasks of road exploration, security patrol, snowplow street clearing and street photography ^6–8^, the main constraints of KCPP are the large-scale road network and the target loop equilibrium. The KCPP problem with constraints on the goal loop equalization is also known as the Min–Max k Chinese Postman Problem (MMKCPP).

MMKCPP, based on KCPP, requires to find k balanced shortest circuits in an undirected graph to ensure the balance of task allocation and improve the efficiency of task completion. Since MMKCPP is an NP-hard problem, the research for this problem is dominated by approximation algorithms. Shuhe Wang proposed a dedicated right-handed passing KPCPP algorithm for right-handed passing environments ^9^, but this algorithm is only for the case where the road conforms to the right-handed passing, and is not generalized. Frederickson GN et al. proposed the approximation algorithm K-POSTMEN ^10^ based on the Eulerian loop construction algorithm of Edmonds and Johnson, which formed k equalization loops by segmentation. However, the loop length obtained by this method is far from the ideal optimal length. Dino Ahr et al. proposed Augment-Merge algorithm ^11^, which is solved by continuously adjusting the longest and shortest loop paths. Subsequently, they introduced the Tabu Search algorithm ^12^, which relies on an initial solution and a moving strategy to find the optimal solution. All of these methods are able to find k equalization loops, but the computational effort increases significantly when facing a large-scale road network. Rong Fei et al. proposed a solution method using dynamic programming ^13–15^, which first interchanges point edges on the road map and constructs a dynamic programming model from it, which is more complex and the overall computational length of the algorithm increases dramatically with the increase of the complexity of the road network. Wei Yu et al. also proposed an improved approximation algorithm, 4-approximation algorithm for MMKCPP ^16–19^, and the equalization loop length obtained by the method is four times of the ideal optimal length.

Most of the existing methods have been studied for small-scale road networks. In the actual task, there often exists the constraint of large-scale road networks, and it is usually difficult for the existing methods to find the balanced multiple paths under large-scale road networks. Meanwhile, the gap between the longest path lengths obtained by existing methods and the ideal optimal value increases with the increase of the road network size. Therefore, in order to solve this difficulty and improve the applicability of the methods to large-scale road networks, this paper studies the MMKCPP problem. A solution method (Critical tabu ant k-routes method, CTA-kroutes) based on the idea of critical edges and using the tabu search algorithm and ant colony algorithm is proposed. The method itself employs heuristic and solution optimization steps, using the tabu search algorithm to continuously search for the optimal solution from within the neighborhood formed by the movement of critical edges. And an ant colony algorithm for solving RPP (Rural Postman Problem, Rural postman problem) is used to optimize the solution for each search.

The remaining part of this article is organized as follows: In Section "MMKCPP problem definition and mathematical model", the MMKCPP problem and its mathematical model are described; In Section "CTA-kroutes algorithmic framework", the CTA-kroutes algorithm and its main contributions are introduced; In Section "Experimental results and analysis", the results and improvements of the CTA-kroutes method are presented; Finally, a summary is provided in Section "Conclusion".

## MMKCPP problem definition and mathematical model

### Problem definition

MMKCPP is defined as follows: given an undirected connected graph G = (V, E), each edge has weights w: E → R^+^, starting point *v*_1_, number of vehicles k (k ≥ 2), find k strip of loops S = (RL_1_, RL_2_, …, RL_k_ ) such that ${\bigcup }_{1\le i\le k}{E(RL}_{i})$ = E(G) and $\underset{1\le i\le k}{\mathit{max}}(L(RLi))$ minimize. where V is the set of vertices, E is the set of edges, RL is the loop, and L(RL) is the path length of the loop.

The loop RL is defined as follows: given a sequence of vertices *v*_1_, *v*_2_, …, *v*_*n*_, such that (*v*_*i*_, *v*_*i*+1_ ) ∈ *E* (1 ≤ *i* < *n*), then the vertex sequence *v*_1_, *v*_2_, …, *v*_*n*_ denotes a path from point *v*_1_ to point *v*_*n*_ . A path is said to be a loop, denoted RL, if *v*_1_ = *v*_*n*_ .

### Mathematical modeling

The set of all possible solutions that satisfy the constraints is denoted as *M*, each S consists of k loops:

$$M=\{{S}_{1},{S}_{2},\dots \}$$

$$S=\{R{L}_{1},R{L}_{2},\dots ,R{L}_{k}\}$$

1. Objective function: find the set of loops whose longest path length is minimized: ${S}_{opt}$
  The length of the longest loop path in the *S*:
  $${L}_{max}\left(S\right)=\underset{\mathit{RL}\in S}{\mathit{max}}({\sum }_{e \in RL}\omega \left(e\right)*{x}_{e})$$
  Optimal solution ${S}_{opt}\epsilon M, {L}_{max}({S}_{opt})$ satisfies:
  $${L}_{max}\left({S}_{opt}\right)= \underset{S\in M}{\mathit{min}}({L}_{max}(S))$$
  where $\omega \left(e\right)$ is the weight of the edge $e$, and ${x}_{e}$ is the number of times the edge $e$ passes through the loop RL.
2. Constraint 1: Each edge is passed through S at least 1 time.
  $${\sum }_{RL \in S}{x}_{e}\ge 1 \forall e\in E$$
3. Constraint 2: The target paths are all loops.
  $${\sum }_{e\in \delta \left(v\right),v\in V(RL)}{x}_{e}\equiv 0 mod 2 \forall RL\in S$$
  where $\delta \left(v\right)$ is the number of sides passing through the point $v$, and $V(RL)$ is the set of points in the loop RL.
4. Constraint 3: The number of traversals per edge should be an integer.
  $${x}_{e}\in \left\{\text{1,2},\dots \right\}\forall e\in E$$
5. Constraint 4: Number of target loops.
  $$Num\left(S\right)=k$$

## CTA-kroutes algorithmic framework

### CTA-kroutes algorithm flow

The basic idea of the CTA-kroutes algorithm is to improve the initial solution by means of a tabu search algorithm, which continuously searches for the optimal solution. The main contribution of this paper is to introduce the idea of critical edges in the construction process of the neighborhood and to propose the corresponding solution optimization method. The CTA-kroutes algorithm has three parts, which include: the construction of the initial solution, the construction of the neighborhood using critical edges and tabu search, and the solution optimization method. The algorithm flow is as follows:

1. The initial solution S1, which is the current solution S, is found using the improved K-POSTMEN algorithm;
2. Compute the set of critical edges KE in the graph G and compute the set of critical edges Sk contained in each loop RL in the initial solution S1;
3. Construct a neighborhood by moving critical edges and search for the optimal solution using a tabu search algorithm, select the next current solution S in the neighborhood according to the tabu table, and record if S is better than the current optimal solution opt;
4. Perform a solve-optimize operation on the current solution S;
5. If the number of iterations without improvement of the optimal solution exceeds the upper limit or the next current solution S cannot be selected in the neighborhood, the algorithm ends; otherwise, jump to 3) to continue the iterative search;
6. Output the optimal solution OPT during the iterative search.

In Section "Initial solution construction" the method of constructing the initial solution will be introduced first. After that, in Section" Tabu search algorithm based on critical edges" the computation of critical edges and how to use critical edges for neighborhood construction and search to improve the initial solution will be introduced respectively. Finally, in Section "Solving the optimization process" how to optimize the search solution will be presented.

### Initial solution construction

**Figure 1:**
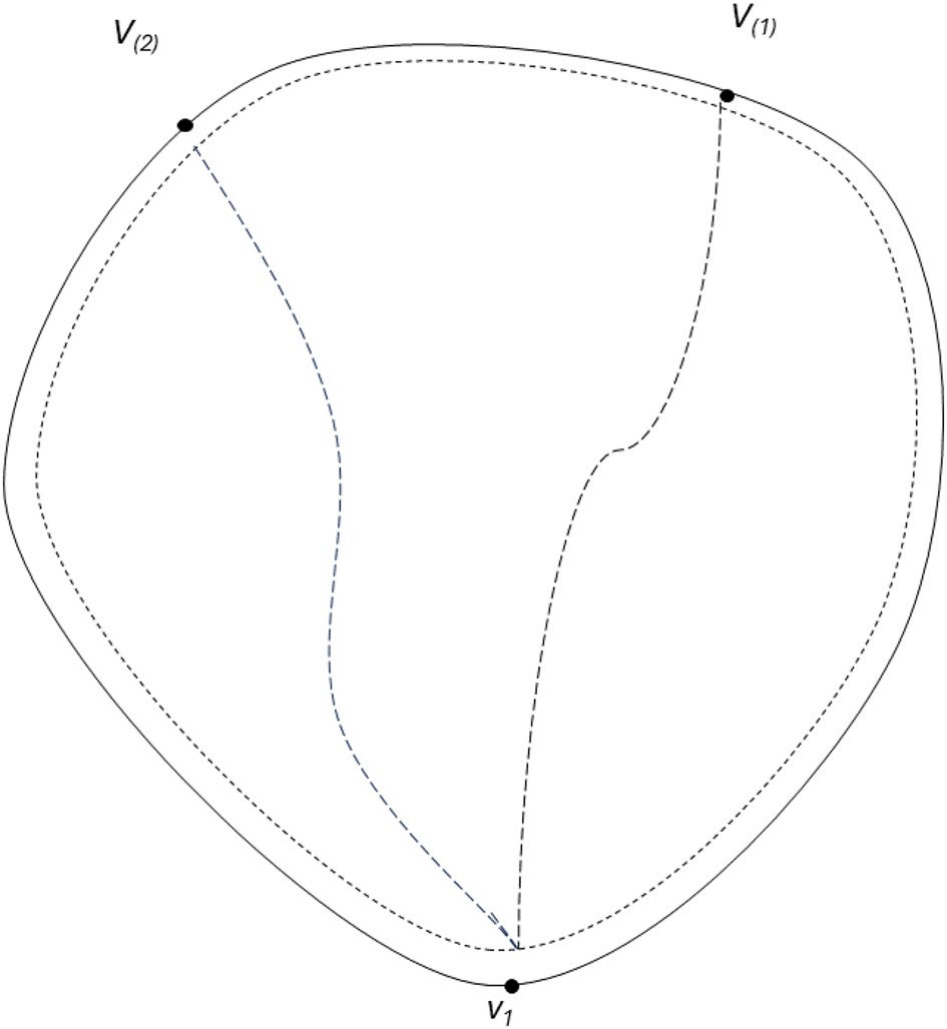
Schematic diagram of the initial solution for k = 3.

The basic idea of CTA-kroutes algorithm is the improvement of the initial solution, and the merit of the initial solution will affect the convergence time of the subsequent tabu search and the reliability of the search results. Therefore, in this paper, we improve on the K-POSTMEN algorithm to construct the initial solution with more balanced length of each loop. The algorithm is procedure 1, where ER = {v_1_ ,v_2_ ,…,v_1_ } is the Eulerian loop on the undirected graph G with starting point v_1_ . This algorithm performs a balanced decomposition of the Eulerian loops, improves the calculation method of each len, and enlarges the value of each midlen appropriately when finding each path, which makes the lengths of the k loops in the initial solution S1 more balanced. the relevant schematic diagram for k = 3 is shown in Fig. 1.

**Procedure 1:**
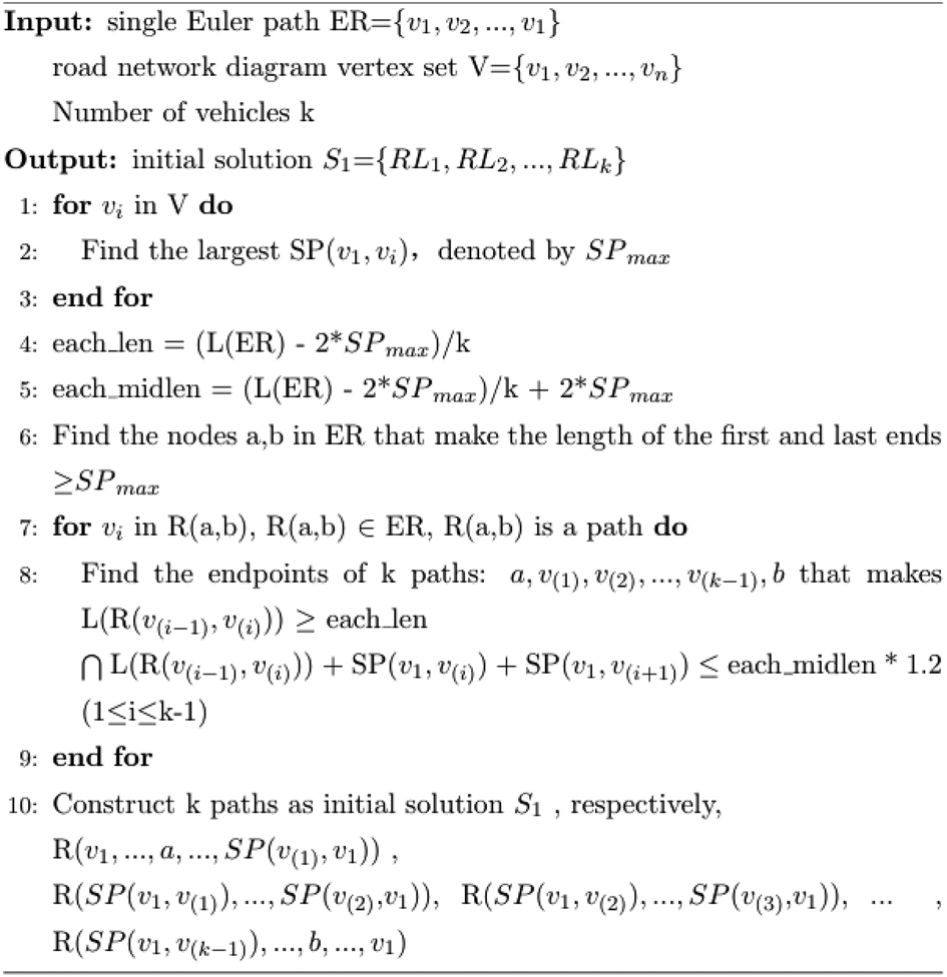
Initial solution construction

In the initial solution construction, each_len is the segmentation length of each loop in the single Euler path segmentation after removing the length of the first and last ends. each_midlen is the total length of each loop in the single Euler path segmentation. As in Fig. 1, each loop can be expressed as SP(v_1_,v_(1)_) + SP(v_1_,v_(2)_) + R(v_(1)_,v_(2)_). SP(v_1_,v_(1)_) and SP(v_1_,v_(2)_) are the paths of the first and last ends of the loop, and R(v_(1)_,v_(2)_) is the path of the loop split. each_len is a criterion for the segmentation length, which is computed by taking the mean of the k paths after subtracting the maximum length of the first and last ends from the total length of the single Euler path. each_midlen is the criterion of the split single loop, which is calculated as each_len plus the maximum length of the length of the first and last ends. The length of the first and last ends is expressed as 2 times SP_max_. SP_max_ is the maximum value of the shortest path from each node in the set of path points to the start point.

### Tabu search algorithm based on critical edges

After the initial solution is constructed, the CTA-kroutes algorithm will improve the initial solution by means of a tabu search algorithm. During the search process, in order to improve the search efficiency and the possibility of convergence of the results to the global optimal solution, the CTA-kroutes algorithm uses critical edges for the construction of the neighborhood. The set of critical edges is a subset of E in the graph G, which contains edges in the graph G that are far from the starting point and discrete. By moving the critical edges in the longest and shortest loops to construct the neighborhood, the effectiveness of each search can be improved and the result can be closer to the global optimal solution.

#### Key edge set computation

In the road network graph G, given the starting point *v*_1_, the shortest path between two vertices *v*_*i*_, *v*_j_ is represented by SP(*v*_*i*_, *v*_j_), and the shortest path can be found by dijkstra's algorithm and floyd's algorithm. For any e ∈ E, LOOP is used to represent the edge loop formed by it and the starting point v_1_, and L(LOOP) denotes the path length of LOOP. All LOOPs form the set LP, LP = {LOOP_1_, LOOP_2_, …, LOOP_m_}. Procedure 2 involves the selection of critical edges and the calculation of LOOP.

1 $$LOOP = e_{ab} + SP\left( {v_{1} , v_{a} } \right) + SP\left( {v_{1} ,v_{b} } \right)\left( {v_{a} ,v_{b} \in V;e_{ab} \in E} \right)$$

Through the above process, the set of critical edges KE = {ke_1_, ke_2_, …, ke_kn_} is formed, which contains the number of critical edges kn.

**Procedure 2:**
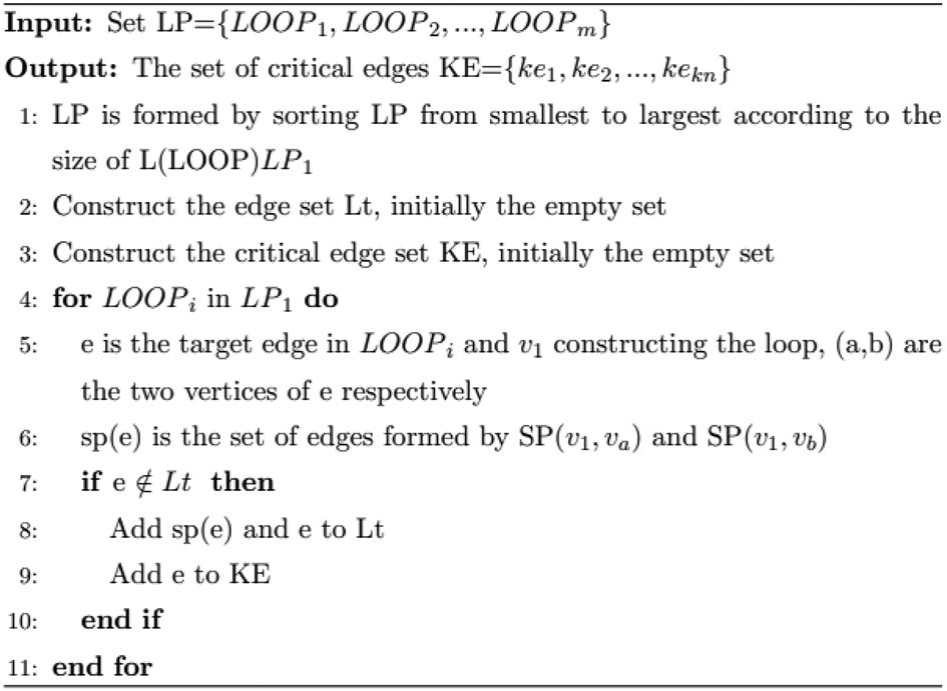
Critical edge selection process

The key edge points out that when the current undirected graph G solves the MMKCPP, the effective number of vehicles k is at most kn . At this point, there are three scenarios of the problem for the given number of vehicles k as follows:

1. When k = kn, the set LP consisting of LOOPs corresponding to each critical edge in KE is the optimal solution of the problem.
2. When k > kn, since kn points out the upper limit of k required for multiple vehicles on the current road network, the solution corresponding to kn remains optimal, and the extra vehicles will only increase the duplicated paths and will not decrease $\text{min}\left(\underset{1\le i\le k}{\text{max}}\left(L\left(R{v}_{i}\right)\right)\right)$ the size of the road network.
3. When k < kn, multiple critical edges are merged to form a new set of critical edges KE1 = {ken_1_, ken_2_, …, ken_k_}, where ken may be a set of multiple ke. At this time, the solution corresponding to KE1 obtained by merging is the final solution. The combination of the critical edges determines the final solution.

The CTA-kroutes algorithm is solved for the case k < kn, and after obtaining the set of critical edges of the undirected graph G, a tabu search algorithm is used for the computation of the near-optimal solution. During the tabu search, the construction of the neighborhood is performed by shifting the critical edges.

#### Neighborhood construction

The core of tabu search is the process of constructing a series of neighborhood solutions by making certain changes to the current solution, selecting the next current solution from them and repeating the process. The neighborhood construction rule determines how the current solution is changed, and also determines the search range and convergence speed of the algorithm. The CTA-kroutes algorithm is based on the critical edges, and moves the critical edges and their peripheral paths, rc, from the longest path, RL_a_, to the shortest path, RL_b_, i.e., removes rc from the longest path, RL_a_, and adds rc to the shortest path, RL_b_ . This construction rule ensures the completeness of solution S, and improves the search speed compared with the scheme of moving each edge. S completeness while improving the search capability of the algorithm compared to the scheme of moving each edge, and accelerates the search efficiency of the algorithm and avoids many meaningless search processes.

**Figure 2:**
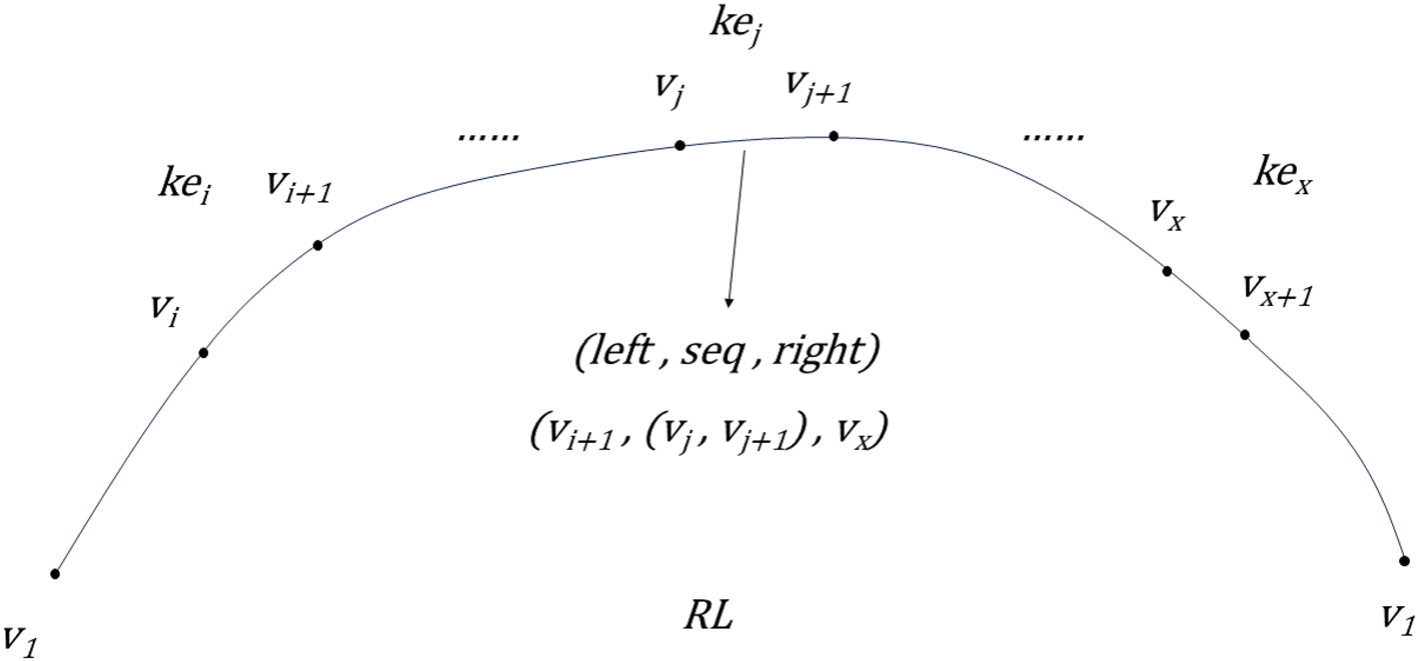
Example of rc and cone.

The tabu unit ‘cone’ is formed by rc, denoted as (left, seq, right), where seq is the number of the critical edge, left is the connected node that is closest to the critical edge to the left of seq under the sequence along the current path RL, and similarly, right is the connected node that is closest to the critical edge to the right of seq under the sequence along the current path RL. As in Fig. 2, the graph has rc = {v_i+1_, …, v_j_, v_j+1_, …, v_x_} and cone = {v_i+1_, (v_j_, v_j+1_), v_x_}.

**Figure 3:**
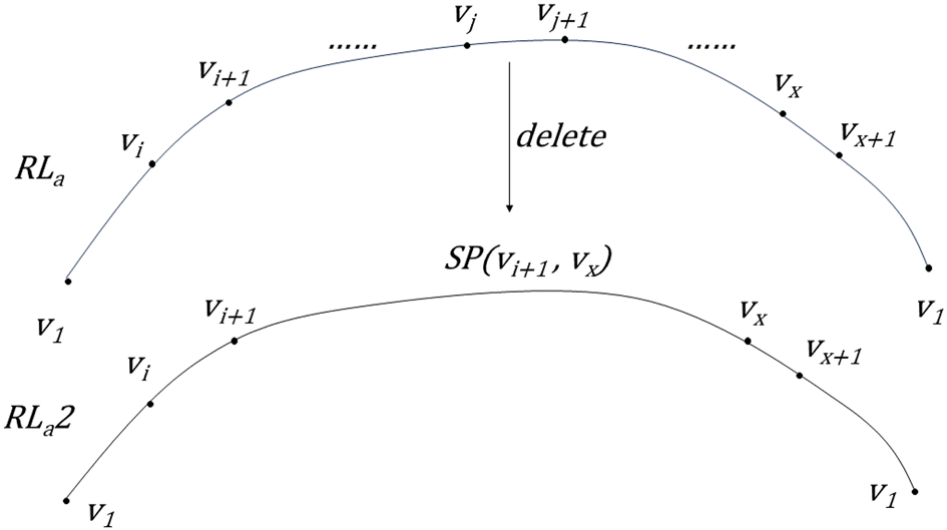
Deletion process of rc.

**Figure 4:**
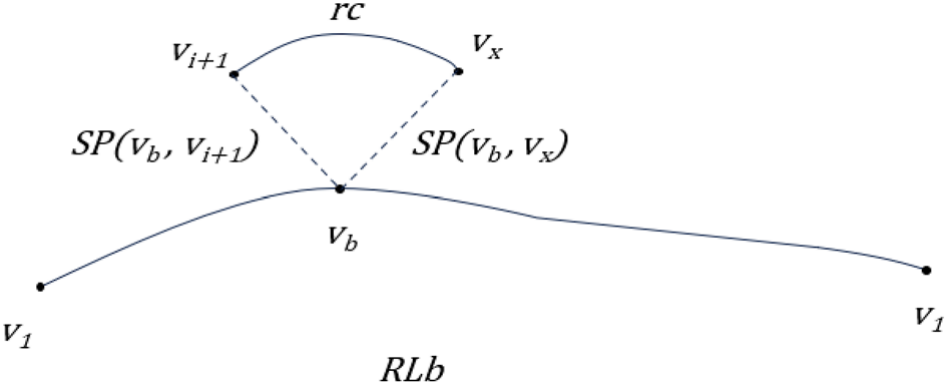
Flow of adding rc.

The process of rc deletion: in order to ensure connectivity, uses SP(v_i+1_, v_x_) in RL_a_ to replace the relevant path of rc and completes the deletion of rc and generates a new RL_a_2. The process is shown in the Fig. 3:

The process of adding rc: to ensure that the change of L(RL_b_ ) is minimized, it is necessary to find the node v_b_ in V(RL_b_) that is closest to the distance between the two endpoints of rc, i.e. ${v}_{b}=\text{min}\left(SP\left({v}_{m},{v}_{i+1}\right)+SP\left({v}_{m},{v}_{x}\right)\right) \left({v}_{m}\in V\left({RL}_{b}\right)\right)$. The flowchart is shown in Fig. 4.

#### Tabu search process

After using the critical edges for neighborhood construction, the tabu search algorithm will select the current optimal solution as the input for the next search according to the tabu table and the generation value of the neighborhood solution, and keep repeating the process of neighborhood construction and the selection of the current optimal solution until the improvement of the optimal solution is not achieved within a certain number of iterations or there is no feasible solution in the neighborhood, the tabu search algorithm ends, and the optimal solution in the search process is the algorithm output. The tabu search process is as follows, where rlc represents the set of critical edges contained in each loop RL in S, the generation value of the neighborhood solution is the amount of change with respect to the current max(S), and ‘cone’ is the unit of the tabu table. opt represents the optimal solution, and max(opt) represents the maximum length in the optimal solution. The algorithm process is procedure 3.

**Procedure 3:**
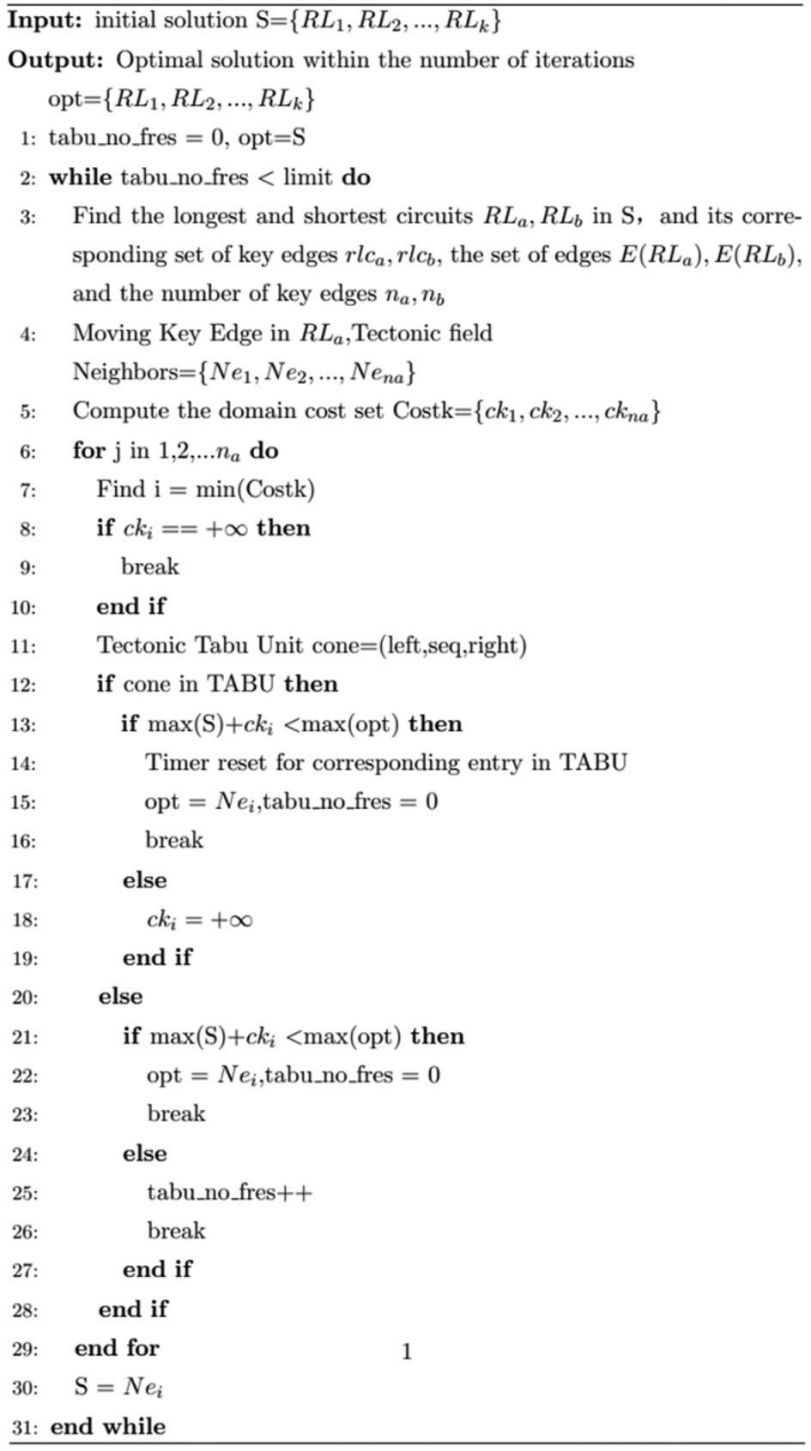
Tabu search process

### Solving the optimization process

Local solutions for each search in tabu search are generated by moving critical edges, and each key edge move causes a change in the allocation of non-critical edges. At the same time, the construction goal of the neighborhood solution is to run fast and represent the surrogate values as accurately as possible. The above reasons lead to the presence of more duplicate edges in the local solution, where the length of each loop path can still be shortened. Therefore, after obtaining the local solution for each search, solution optimization is required to reduce the number of duplicate edges and also to optimize the structure of the solution using the yq_RPP algorithm to generate new loops. The process of solution optimization is as shown in procedure 4.

**Procedure 4:**
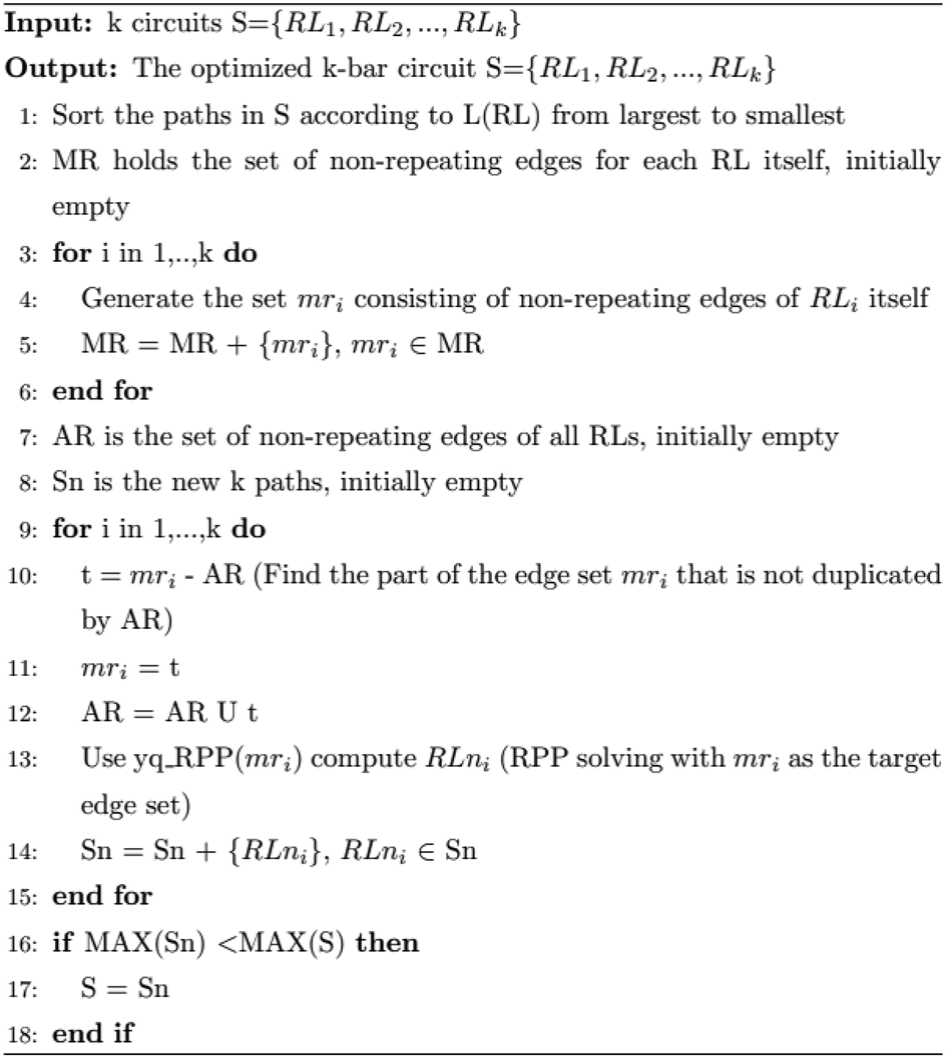
Solving the optimization process

The main role of the solution optimization is to remove the duplicate edges of each loop and calculate the new loop after removing the duplicate edges. The solution optimization algorithm can effectively reduce the length of each path, improve the effectiveness of the subsequent search, and more conducive to the convergence of the search to the optimal solution.

## Experimental results and analysis

In this paper, experiments are conducted in Win10 platform and Visual Studio 2019 environment. The inputs are undirected connected road network graphs of different sizes, in which the path lengths between nodes are randomly generated. This is done in order to simulate the randomness between different path lengths in the real road network environment, so as to exclude the error caused by the specificity of the data on the experimental results. The outputs are the K-POSTMEN algorithm, the tabu algorithm and CTA-kroutes algorithms to calculate the maximum path length in k loops.

The percentage of difference between the longest path length and the ideal optimal solution as well as the approximation ratio are used as evaluation metrics in the experiments. For the comparison object, K-POSTMEN algorithm is used for the traditional algorithm and tabu algorithm is used for the heuristic algorithm. The rest of the proposed algorithms include deterministic algorithms as well as approximation algorithms. For deterministic algorithms, the comparison is not relevant due to the unacceptable computation time at larger scale. For approximate algorithms, the approximation ratio in certain cases is provided as a reference due to the variation in the solution of the heuristic algorithm. The study mainly aims at improving the feasible solution, expecting to obtain a more superior feasible solution in an acceptable time. Therefore, the computational time of the algorithm has not been improved and the computational time remains flat compared to the tabu algorithm and longer than the K-POSTMEN algorithm. The computational time of the algorithm is not the direction of improvement or innovation in this paper, so subsequent experiments will focus on the comparison of results.

### Comparison of algorithm results for different number of vehicles k

In the experiment, the mean score of the total path length under different scales for k is used as the ideal optimal solution ideal-opt for MMKCPP under the current scale road network, and this value is the upper bound and the true optimal solution true-opt must be greater than or equal to the ideal-opt. A total of three scales of road networks are set up in the experiment, whose node numbers and edge numbers are (20,190), (40,780), (60,1770), and the MMKCPP problem is solved using different algorithms for the above three sizes of road networks under k = 2, 5, 10, 15, and 20.

The solution results are as follows, where Ki, ti, Ci denote, respectively, the difference between the results of the K-POSTMEN algorithm, the tabu algorithm, and the CTA-kroutes algorithm and ideal-opt. Tabu algorithm do not use a solution optimization step and critical edge construction. Ideal denotes ideal-opt, K-P denotes K-POSTMEN. CTA denotes CTA-kroutes. path units are all m. The percentage of difference relative to the ideal optimal solution is calculated as in Eq. (2).

2 $$Mi=\frac{M-ideal}{ideal}*100\text{\%}$$

where Mi denotes the difference of algorithm M with respect to ideal-opt, M denotes the comparison algorithm, and ideal denotes the ideal optimal solution.

**Figure 5:**
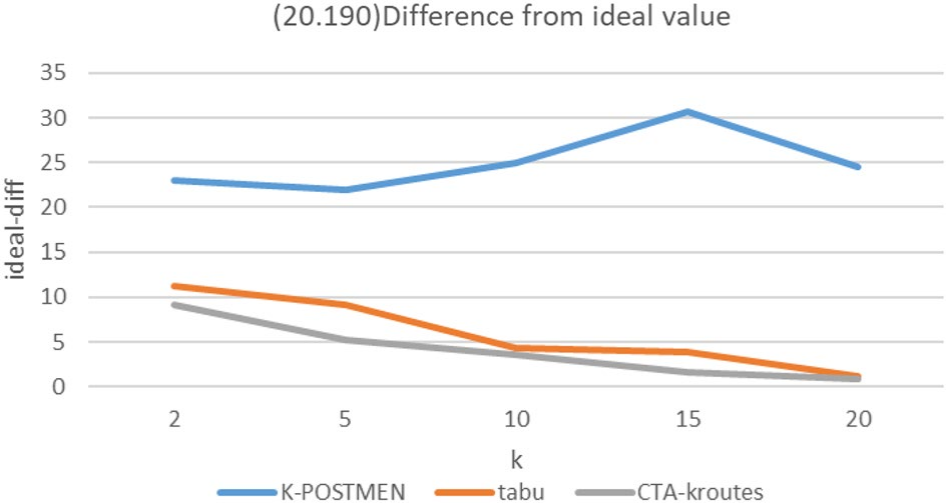
(20.190) Difference from ideal value.

**Figure 6:**
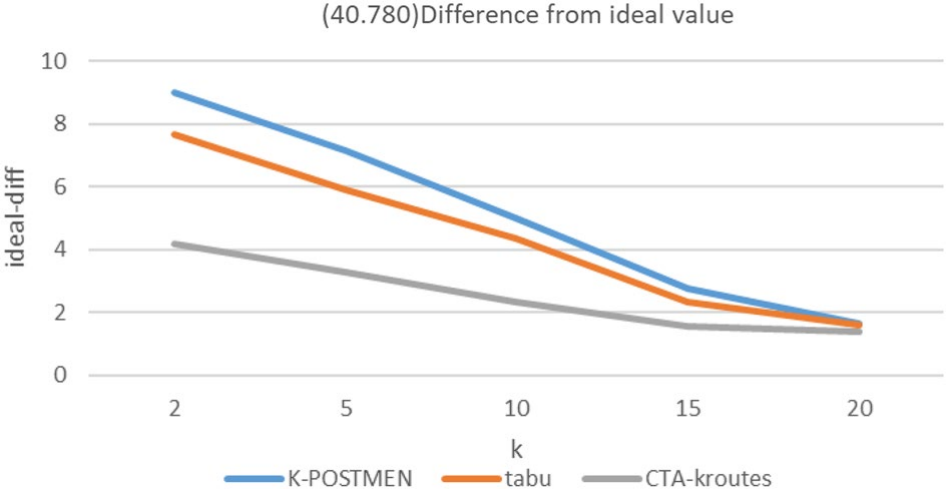
(40,780) Difference from ideal value.

**Figure 7:**
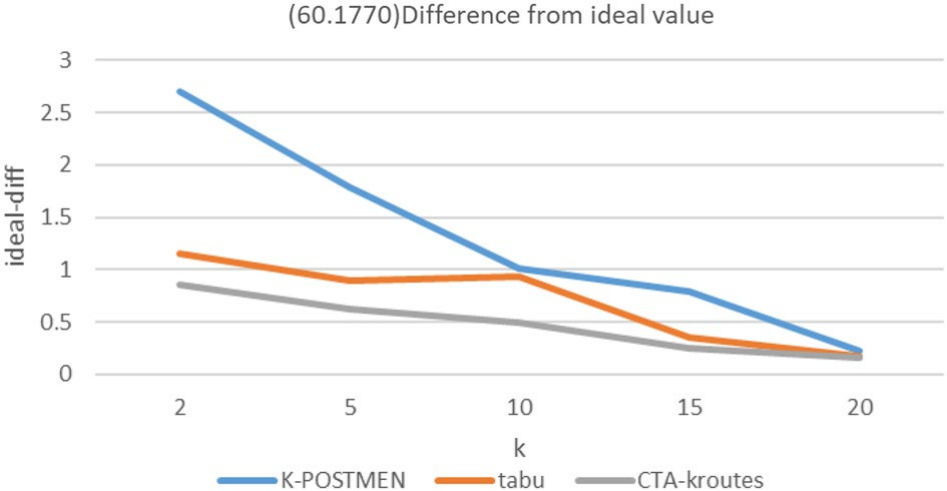
(60,1770) Difference from ideal value.

From the Figs. 5, 6 and 7, it can be seen that the CTA-kroutes algorithm improves compared to both K-POSTMEN and tabu algorithms under different sizes of road network and different number of vehicles k. Since the reference is IDEAL-OPT and not actual TRUE-OPT, the enhancement is not fixed in some cases.

The results of MMKCPP are related to the balance of the length of each edge in the road network diagram, the length of each edge and the number of vehicles. The more balanced and shorter the length of each edge in the road network graph, the easier it is to solve the feasible solution of MMKCPP. Additionally, the closer the feasible solution is to the ideal optimal solution. At this time, the gap between the algorithms is smaller. At the same time, the more the number of vehicles, the smaller the ideal optimal value, the more difficult to reflect the gap between different algorithms. Figure 5 reflects the improvement effect of tabu search compared with the traditional method. Figure 6 reflects the enhancement effect of the tabu search combined with key edges compared with the traditional tabu search. And the construction of the neighborhood combined with key edges further improves the quality of the feasible solution obtained by the tabu. search algorithm and improves the effectiveness of the feasible solution. Figure 7 simulates the case of balanced edge lengths and shorter edge lengths in the road network graph, when the solution obtained by the algorithm is still closer to the ideal solution.

### Comparison of different algorithms for boosting

The lift of the algorithm is benchmarked against the K-POSTMEN algorithm and the tabu algorithm, and the lift is the difference between the algorithm and ideal-opt with respect to the benchmark algorithm. It is calculated as in Eq. (3):

3 $$improve=\frac{Ni-Mi}{Ni}*100\%$$

where Mi denotes the difference of a particular algorithm with respect to ideal-opt, Ni denotes the benchmark algorithm, and improve denotes the magnitude of the improvement.

The lift results are as follows, Ci-Ki denotes the lift of the CTA-kroutes algorithm with respect to the K-POSTMEN algorithm, ti-Ki denotes the lift of the tabu algorithm with respect to the K-POSTMEN algorithm, and Ci-ti denotes the lift of the CTA-kroutes algorithm with respect to the tabu algorithm. The lift values are all percentages, and average indicates the average lift of the algorithm at the current scale (Table 1).

**Table 1:** a(20, 190), b(40, 780), c(60, 1770) Lift under scale road network.

k	a.ti-Ki	a.Ci-Ki	a.Ci-ti	b.ti-Ki	b.Ci-Ki	b.Ci-ti	c.ti-Ki	c.Ci-Ki	c.Ci-ti
2	26.08	43.47	17.39	2.45	17.29	14.83	22.58	32.25	9.67
5	4.54	59.09	54.54	15.11	43.36	28.24	56.07	67.75	11.68
10	52.00	60.00	8.00	13.14	52.96	39.81	7.29	51.09	43.79
15	45.60	68.40	22.80	17.84	54.47	36.62	49.58	65.07	15.49
20	36.73	48.97	12.24	14.96	53.79	38.82	57.22	68.11	10.89
average	32.99	55.99	22.99	12.70	44.37	31.67	38.55	56.86	18.30

In all the experiments, the algorithms were boosted on average, as shown in Table 2.

**Table 2:** Total average algorithmic lift.

ti-Ki	28.08
Ci-Ki	52.40
Ci-ti	24.32

From the Table 1, it can be seen that the initial solution construction, tabu search, and solution optimization methods adopted by CTA-kroutes can effectively reduce the length of the longest loop of the optimal solution and make it closer to the ideal optimal solution.

### Algorithm Approximation Ratio Analysis

Since the CTA-kroutes algorithm takes the K-POSTMEN algorithm as the initial solution and improves upon it, the worst case of the solution obtained by the CTA-kroutes algorithm in general is shown in Eq. (4). Therefore, the CTA-kroutes algorithm is a 4-approximation algorithm, i.e., the worst case of the solution obtained by it is four times the ideal-opt. In the current optimal case, the solution obtained by the CTA-kroutes algorithm is 1.01 times the ideal-opt.

4 $$\text{max}\left(CTA-kroutes\right)=\frac{4x}{k}$$

where $\text{max}\left(CTA-kroutes\right)$ is the longest path length in the CTA-kroutes result, x is the total path length of the original road network, k is the number of postmans, and x/k is the ideal-opt.

The main body of the CTA-kroutes algorithm is divided into two steps: construction of the initial solution and optimization of the k loops. The construction of the initial solution includes the construction of the Euler loop and the construction of the k initial loops. The simplest Euler loop construction method is to add a duplicate edge to all edges of the original road network. This method is the worst case of Euler graph construction, the length of the Euler loop is 2x. From the construction method of each_len in the initial structure, the worst case of each_len is $2x/k$. At this time, in the process of each_midlen, when ${SP}_{max}\le x/k$ he worst case of the CTA-kroutes is $4x/k$. when ${SP}_{max}>x/k$, the worst case of ${SP}_{max}$ is x, i.e., all the edges are on a straight line, and the worst case is $(\left(2+2k\right)x)/k$. However, in general, the ${SP}_{max}$ in the road network graph won't be more than $x/k$, so we take $4x/k$ as the approximation of the worst case of CTA-kroutes here.

## Conclusion

In this paper, a heuristic algorithm CTA-kroutes for solving the min–max multi-vehicle Chinese letter carrier problem is proposed by combining the tabu search algorithm and the key edge idea. The method searches for the optimal allocation scheme of the key edges in the initial solution within the iteration number through the tabu search algorithm, and proposes the yq_RPP algorithm for solving the RPP formed after the allocation of key edges. The neighborhood construction scheme and tabu table update scheme proposed in this paper effectively reduce the occurrence of invalid iterations. This enables the method to find the feasible solution faster within the specified number of iterations, even for large-scale road networks. As a result, the availability of results is ensured.

In the experimental results, the usability of the solutions obtained by the CTA-kroutes algorithm is improved for different sizes of road networks. Thus, it shows that the algorithm is optimized in the computation of the solution as well as in the construction of the neighborhood compared to the traditional algorithm K-POSTMEN and the heuristic algorithm tabu. Since the evaluation criterion of MMKCPP is the proximity to the ideal optimal solution, it is easier to see in the performance comparison among the algorithms that the CTA-kroutes algorithm has improvement in the effectiveness of the solution. Finally, the approximation ratio of the algorithm in the case of ${SP}_{max}\le \frac{x}{k}$ also represents the upper limit of the solution of CTA-kroutes algorithm in the worst case, which ensures the applicability of the algorithm. The large scale of the road network for all the experiments in this paper ensures the usability of the algorithm on large-scale road networks.

## Data Availability

The datasets analysed during the current study are available from the corresponding author on reasonable request.
